# Association of Serum Creatinine, Urea, and Glomerular Filtration Rate with the Progression of Diabetic Associated Kidney Complications: A Retrospective Case-Control Study

**DOI:** 10.3390/cimb48020167

**Published:** 2026-02-02

**Authors:** Shahad Saif Khandker, Shoumik Kundu, Farhana Ahmed, Adiba Ayesha Khan, Lamiya Farhin, Farhana Islam, Rahima Begum, Md Jasim Uddin, A. N. M. Mamun-Or-Rashid

**Affiliations:** 1 Department of Biochemistry and Molecular Biology, Gono Bishwabidyalay, Dhaka 1344, Bangladesh; shahadsaifkhandker@gmail.com; 2 Department of Chemistry and Biochemistry, Texas Tech University, 2500 Broadway W, Lubbock, TX 79409, USA; shoumikk33@gmail.com; 3 Department of Pharmacy, East West University, Dhaka 1212, Bangladesh; farhanaahmed.6619@gmail.com; 4 School of Pharmacy, BRAC University, Dhaka 1212, Bangladesh; adiba.ayesha.khan.aban@gmail.com (A.A.K.); farhinlamiya88@gmail.com (L.F.); 5 Department of Pharmacy, Jashore University of Science and Technology, Jashore 7408, Bangladesh; fa.islam@just.edu.bd; 6 Department of Microbiology, Gono Bishwabidyalay, Dhaka 1344, Bangladesh; 7 Department of Biochemistry and Molecular Biology, Jahangirnagar University, Dhaka 1342, Bangladesh; jasimhabib1994@gmail.com; 8 Department of Environmental and Occupational Health, University of Pittsburgh, Pittsburgh, PA 15261, USA

**Keywords:** diabetic nephropathy, renal function, kidney, diabetes, biomarkers, protein

## Abstract

Introduction: Diabetes mellitus (DM) is a prevalent metabolic disorder frequently leading to serious renal complications, particularly diabetic nephropathy. This retrospective case–control study investigated the levels and associations of commonly used enzymatic (serum creatinine and urea) and physiological (glomerular filtration rate [GFR]) markers of kidney function in diabetic patients compared to non-diabetic controls. Methodology: A total of 237 participants were enrolled, comprising 81 diabetic cases and 156 non-diabetic controls. Creatinine and urea levels were determined using enzymatic methods, measuring optical density, whereas GFR was calculated using the Chronic Kidney Disease Epidemiology Collaboration (CKD-EPI) equation, based on creatinine, age, and sex. Statistical comparisons include *p*-value, Pearson correlation, etc. Results: The diabetic group exhibited significantly higher mean levels of serum creatinine (2.08 ± 2.26 mg/dL) and urea (57.71 ± 38.75 mg/dL) and a significantly lower mean GFR (59.59 ± 34.16 mL/min/1.73 m^2^) compared to the non-diabetic control group (0.95 ± 0.69 mg/dL, 31.79 ± 20.49 mg/dL, and 96.72 ± 23.77 mL/min/1.73 m^2^, respectively; all comparisons with *p* < 0.005). Correlation analysis revealed a more scattered positive association between creatinine and urea, and a pronounced inverse correlation between GFR and both creatinine and urea in the diabetic cases, suggesting a compromised renal function profile. Conclusions: Our findings demonstrate a significant association between diabetes and impaired renal function, as evidenced by elevated creatinine and urea levels and reduced GFR. These readily available biomarkers are crucial prognostic indicators for the early detection and effective management of diabetic nephropathy, emphasizing the importance of rigorous metabolic and blood pressure control to mitigate disease progression.

## 1. Introduction

Diabetes is one of the most common chronic non-communicable diseases and serious health issues worldwide, with its occurrence steadily on the rise [1]. Epidemiological data indicate that the escalating incidence of diabetes and its associated complications remain a critical and continuously growing public health concern worldwide. In 2021, approximately 10% of the global adult population, equating to about 537 million individuals, were living with diabetes, a number projected to reach approximately 600 million by 2035 [2,3]. According to the study of GBD 2021 Diabetes Collaborators, diabetes will affect 1.3 billion people by 2050, with prevalence anticipated to rise significantly in low- and middle-income countries (LMICs) [4]. An analysis between 2000 and 2019 showed a 3% rise in age-standardized mortality rates attributed to diabetes [4]. This chronic condition not only impacts individuals’ daily lives but also imposes substantial economic burdens on global healthcare systems. Effective management and prevention strategies are therefore crucial to alleviate the growing impact of diabetes on public health [2,5].

Diabetic patients face a heightened risk of developing severe complications such as nephropathy, retinopathy, neuropathy, and atherosclerosis. Notably, approximately one-third of individuals with type 2 diabetes develop diabetic nephropathy [6]. Diabetic nephropathy (DN) is a complex disorder characterized by a range of interconnected processes, with hyperglycemia being the primary influencing factor. This disorder emerges from multiple factors, involving metabolic disturbances from high blood sugar, changes in blood flow, inflammatory reactions, oxidative stress, and genetic predisposition. Key mechanisms include advanced glycation end products (AGEs) formation, excessive renin–angiotensin–aldosterone system (RAAS) activity, and protein kinase C (PKC) activation. These processes lead to increased glomerular filtration, enlargement, kidney tissue fibrosis, and scarring. High blood sugar levels cause mesangial cell proliferation and glomerular basement membrane thickening in early DN stages, resulting in progressive glomerular sclerosis and tubulointerstitial fibrosis [7].

Serum creatinine is a crucial biomarker and an important indicator for assessing overall kidney function. However, glomerular filtration rate (GFR) is usually considered one of the most useful markers of renal excretory function [8]. Serum urea on the other hand, is a prognostic indicator and predictor of renal impairment [9]. Elevated levels of both blood urea and serum creatinine suggest prolonged hyperglycemia, which can lead to irreversible damage to the kidney nephrons. This hyperglycemic condition can further disrupt fluid balance, which may ultimately trigger nephropathy [10]. Thus, serum creatinine, urea, and GFR are the crucial biomarkers to identify nephropathy, especially associated with diabetes. In addition, these three biomarkers are readily available biomarkers for renal function assessment in most diagnostic and clinical settings globally [11]. Therefore, understanding their association with renal and kidney diseases is vital to enable clinicians to easily identify and strongly recognize potential renal complications in both symptomatic and asymptomatic diabetic patients.

Unfortunately, there is a distinct lack of comprehensive data on the plasma biochemical profiles—specifically indicating kidney complications, dysfunction, or nephropathy—among the diabetic population in Pakistan. Furthermore, no study compared these three markers in assessing diabetic-associated nephropathy in the diabetic population compared to controls. A detailed investigation and comparison of these kidney biomarkers between diabetic and non-diabetic subjects could provide valuable insight into this phenomenon. Consequently, this study aimed to compare serum creatinine levels, urea, and GFR between diabetic patients and non-diabetic control residents in Pakistan.

## 2. Method and Materials

### 2.1. Study Settings and Participants

The study was a retrospective case–control design. Data were collected from the records of Abbas Institute of Medical Sciences, Muzaffarabad, Azad Kashmir, Pakistan. The data collection and analysis period spanned from August 2024 to December 2024.

As we considered a specific period of five months (i.e., August to December), within this period, 108 patients came to our hospital who had only diabetes and no other diagnosed disease. Therefore, the total population size was *n* = 108. Among them, 81 patients consented to participate, although with a 95% CI, 5% margin of error, and a 50% response distribution, the sample size should have been *n* = 85. However, if we consider a 90% CI, then *n* = 78 is enough as the appropriate sample size. Therefore, we included 81 participants who consented in our case group. In addition, according to a previous study, 1:2 or 1:4 ratio can be obtained in case–control studies [12]. Therefore, we consider 1:2 ratio as control participants were much more available as compared to cases within that specific study period. Finally, a total of 237 participants were included in the study: 81 constituted the diabetic case group, and 156 comprised the non-diabetic control group. The sample size was calculated using http://www.raosoft.com/samplesize.html (accessed on 1 January 2025), and the calculation method was obtained from a previous study with slight modifications [13].

### 2.2. Ethics Approval and Consent to Participate

Informed consent for participation and data collection was obtained from all included subjects. As this was a retrospective study involving no intervention, compliance with the Declaration of Helsinki was strictly maintained regarding data privacy. The study protocol received approval from the Ethical Committee of AIMS Muzaffarabad, Pakistan (1268/AIMS/2024). This was collaborative research work between Bangladesh and Pakistan.

### 2.3. Inclusion and Exclusion Criteria

Participants were selected based on specific criteria. The inclusion criterion for the case group was a diagnosis of Type 2 Diabetes Mellitus (T2DM). Although all the patients (cases) were previously diagnosed with T2DM in other hospital settings, they were further tested in our hospital settings to reconfirm the T2DM. The control group consisted of participants presenting for random health check-ups with no prior diagnosis of diabetes or other specified chronic diseases. Both male and female participants were deemed eligible, and no age restriction was applied. However, as no children and/or adolescent patients visited the hospital, no children and adolescents were included in this study. Exclusion criteria for the case group were the presence of any other confirmed diagnosed diseases besides diabetes, such as any type of metabolic, pathological, hematological, genetic, or organ-specific diseases, as well as pregnancy, malignancy, and autoimmune rheumatic disorders. For the control group, confirmed diagnosed diabetes resulted in exclusion.

### 2.4. Sample and Data Collection

Blood samples for biochemical testing were collected via venipuncture, followed by serum separation from the hematocrit using centrifugation. Serum samples were tested immediately to ensure result quality. If immediate testing was not possible, the serum was stored at −20 °C and thawed carefully before analysis. Additionally, clinical history and other relevant data were collected from both the printed and computerized register records. A blinded manual cross-check was performed to ensure the accuracy of data collection.

### 2.5. Biomarker Evaluation

Serum creatinine and urea levels were assessed using the Abbott Architect System with specific reagent kits. Creatinine was measured based on the kinetic alkaline picrate method [14], while urea was measured using enzymatic testing based on urease [15]. The final concentration measurements for both creatinine and urea were analyzed via spectrophotometry, with optical density (OD) measured at 490 nm for creatinine and 625 nm for urea. Reference ranges were as follows: serum creatinine was <0.55 mg/dL (low) to >1.02 mg/dL (high) for females, and <0.73 mg/dL (low) to >1.18 mg/dL (high) for males. For urea, the normal range was defined as 10–50 mg/dL, with <10 mg/dL as low and >50 mg/dL as high. To maintain test quality, blank samples were used for both tests, pipettes were calibrated and checked, and sampling and tests were performed separately and blindly by at least two different lab personnel.

### 2.6. GFR Assessment

The estimated GFR was calculated using the Chronic Kidney Disease Epidemiology Collaboration (CKD-EPI) creatinine equation (2021), as this equation is advanced, more efficient, and has almost no race-dependent calculative variation. The variables used were mainly creatinine, gender, and age [16]. The reference range for GFR was categorized as <60 mL/min/1.73 m^2^ (low or abnormal) and >60 mL/min/1.73 m^2^ (normal).

### 2.7. Statistical Analysis

Statistical analyses were performed to determine the correlation and significance among creatinine, urea, and GFR. One-sample *t*-tests were used to obtain mean, standard deviation (SD), and *p*-values to investigate the significance of marker differences between diabetic cases and non-diabetic controls using SPSS software (IBM SPSS Statistics, version 27) and Microsoft Excel 365 [11,14]. The normality of data distribution was assessed using the Shapiro–Wilk test to determine the median and interquartile range (IQR), along with further observation of Q-Q plots and histograms. Given the limited sample size, a *p*-value cutoff of <0.005 was selected to measure high and stricter statistical significance and mitigate plausible biases [14]. Scatter dot plots were generated to visualize the relationships among the biomarkers, employing linear trendlines for the correlation type. In addition, Pearson correlation and significance were analyzed for every scatter plot. Furthermore, multivariate analyses were also performed among markers to investigate any further significant association. Statistical analysis was conducted based on previous studies with slight modifications [11,14].

## 3. Results

### 3.1. Participants’ Demographics

A total of 237 participants were included in this study. General characteristics and demographics of the overall participants, including the distribution by gender, as well as the mean ± standard deviation (SD) and median values of age, are elaborated in Table 1.

Here, OGTT denotes “oral glucose tolerance test”; the reference value of normal OGTT is <100 mg/dL in fasting, and <140 mg/dL after 2 h. HbA1c denotes “Hemoglobin A1c or glycated hemoglobin”; the reference value of normal HbA1c is <5.7%, and >6.4%. Age, OGTT fasting, OGTT after 2 h, and HbA1c were expressed as mean ± SD. *p*-value <0.05 implies significant, creatinine, urea, and GFR values are expressed as mean ± SD. The normal range of serum creatinine is 0.73–1.18 mg/dL (male) and 0.55–1.02 mg/dL (female), the normal range of serum urea is 10–50 mg/dL, and the normal range of GFR is >60 mL/min/1.72 m^2^. IQR = interquartile range.

### 3.2. Contrast of Biomarker Levels and GFR

The levels of serum creatinine and urea (biomarkers) and GFR (physiological marker) were compared between diabetic and non-diabetic participants.

Serum Creatinine: The mean level was significantly higher in diabetic cases (2.08 ± 2.27 mg/dL) compared to non-diabetic controls (0.95 ± 0.69 mg/dL).

Serum Urea: The mean level was also significantly higher in the diabetic group (57.71 ± 38.75 mg/dL) compared to the non-diabetic control (31.79 ± 20.56 mg/dL).

GFR: An inverse relationship was observed, with GFR significantly lower in the diabetic case group (59.03 ± 34.20 mL/min/1.73 m^2^) compared to non-diabetic controls (96.72 ± 23.85 mL/min/1.73 m^2^).

The median and IQR values further supported these inter-group variations. The values of median and IQR were determined as 1.80 (1.15–2.20), 56.0 (41.0–75.0), and 59.0 (35.0–78.0) for the creatinine, urea and GFR of diabetic case group and 0.95 (0.67–1.19), 32.0 (23.0–43.0), and 97.0 (78.0–116.0) for creatinine, urea, and GFR of non-diabetic control group, respectively. According to the independent sample *t*-test, all comparisons were determined to be highly significant (*p* < 0.005). In the case of males and females, creatinine level and GFR were similar among the diabetic case group; however, urea levels were slightly higher in females compared to males. On the other hand, males had higher creatinine and serum levels as compared to females, whereas GFR was lower in males than in females in the non-diabetic control group (Table 1). Furthermore, the distribution of all the samples for each variable was determined to be normal by analyzing the Q-Q plots and histograms. However, multivariate analyses among the biomarkers of the two groups did not find any significance (Supplementary Figures S1–S3).

### 3.3. Association of Biomarkers, GFR, and HbA1c

**Figure 1:**
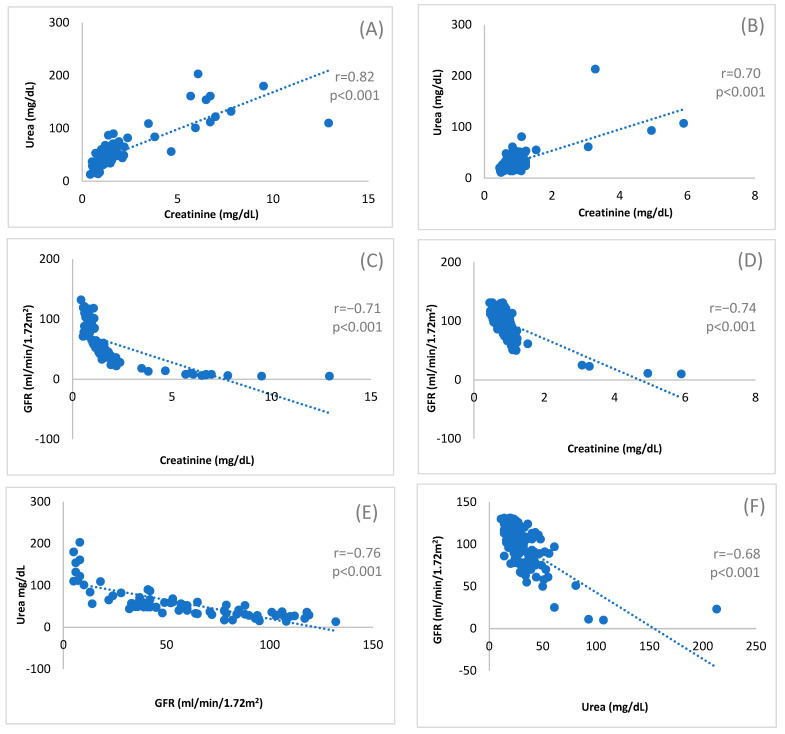
Correlations of serum creatinine and urea, serum creatinine and GFR, and serum urea and GFR between diabetic cases (**A**,**C**,**E**) and non-diabetic control (**B**,**D**,**F**). Plots resemble the association between creatinine and urea (**A**), creatinine and GFR (**C**), and urea and GFR (**E**) in the diabetic case group and the association between creatinine and urea (**B**), creatinine and GFR (**D**), and urea and GFR (**F**) in non-diabetic control group, respectively. *p*-value indicates the significance, and r denotes the correlation coefficient value based on the Pearson correlation.

The association between serum creatinine and urea, serum creatinine and GFR, and urea and GFR was investigated using scatter dot plots (Figure 1).

Diabetic Cases (Figure 1A,C,E): A scattered positive correlation was observed between creatinine and urea. A negative correlation was found between creatinine and GFR, as well as between urea and GFR.

Non-Diabetic Controls (Figure 1B,D,F): The correlation patterns were similar to the diabetic group but were notably less scattered and more concentrated (homogenized) across all three pairs: creatinine and urea, creatinine and GFR, and urea and GFR.

**Figure 2:**
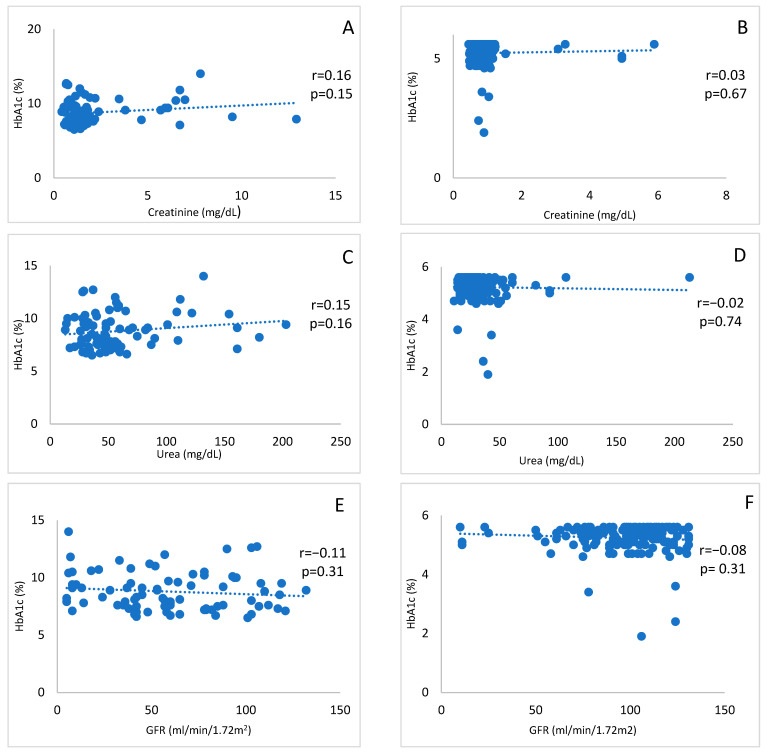
Correlations of serum creatinine, urea, and GFR with HbA1c (**A**,**C**,**E**) of the diabetic (case) group and non-diabetic (control) group (**B**,**D**,**F**), respectively. *p*-value indicates the significance, and r denotes the correlation coefficient value based on the Pearson correlation.

Diabetic Cases (Figure 2A,C,E): Correlation patterns of serum creatinine, urea, and GFR with HbA1c were observed, where positive associations were determined between creatinine and HbA1c, and urea and HbA1c. However, inverse correlation was observed between GFR and HbA1c. Interestingly, no statistically significant correlations were found.

Non-Diabetic Controls (Figure 2B,D,F): While comparing the association of serum creatinine, urea, and GFR with HbA1c, a positive correlation was observed between creatinine and HbA1c, whereas a negative correlation was observed between GFR and HbA1c. Interestingly, in the case of serum creatinine and HbA1c, a slightly positive correlation was observed; however, none of the correlations were statistically significant.

## 4. Discussion

This study primarily identified the significant variation in the level of serum creatinine, urea, and GFR in diabetic patients as compared to their levels in non-diabetic patients (Table 1). It further determined the significant association between each of the two individual markers of each group (Figure 1). It also determined that within the diabetic group, the variation in the levels of these biomarkers in male and female participants is similar, except for the level of serum urea (female > male); however, in the non-diabetic group, males had higher creatinine and urea levels and lower GFR as compared to females (Table 1). No significant association was assessed between each marker and HbA1c level in respective groups (Figure 2). Previous researchers worked on either one or two renal or other markers of diabetic patients and control, where the sample size was smaller (i.e., *n* = 100) including case and control or used only biomarker based ratio (i.e., uric acid/creatinine) [17,18]. However, this study includes three separate renal markers and analyzed association or variation among themselves, between their levels in diabetic and non-diabetic groups, and with a diabetic marker (i.e., HbA1c level). Blood markers such as creatinine and urea are established indicators of renal function impairment, particularly in individuals with diabetes [19,20]. Elevated levels of these markers commonly signal compromised kidney function [21,22]. This study specifically explored the correlation between urea, creatinine, and GFR status when contrasting diabetic cases with non-diabetic controls.

Renal function impairment secondary to diabetes is effectively evaluated by measuring serum urea and creatinine levels, as these biochemical parameters are critical health indicators. Elevated serum levels of both urea and creatinine strongly suggest that the kidneys are operating inefficiently. Regular monitoring of these markers in diabetic patients facilitates the early detection of renal impairment, enabling timely interventions that can potentially slow the progression of renal disease and improve patient prognosis [12]. According to the 2020 Kidney Disease Improving Global Outcomes (KDIGO) Guideline for CKD management, CKD severity is classified into five GFR categories (mL/min/1.73 m^2^). G1 (≥90 mL/min/1.73 m^2^) signifies normal or high kidney function. In contrast, the G3a category (45–59 mL/min/1.73 m^2^) represents a mild to moderate decrease in kidney function, necessitating regular monitoring and potential therapeutic or lifestyle adjustments [23]. Our study demonstrates a significant difference in mean GFR levels between the diabetic and non-diabetic control groups, with a highly significant *p*-value. The non-diabetic controls generally exhibit GFR values within the reference range (≥90 mL/min/1.73 m^2^), placing them in the G1 category. Conversely, the mean GFR of the diabetic group falls into the G3a category (45–59 mL/min/1.73 m^2^), indicating a mild to moderate decline in kidney function. This result strongly highlights the detrimental impact of diabetes on renal health, where lower GFR is a critical marker of compromised renal function in diabetic individuals.

Furthermore, diabetic cases showed significantly higher serum creatinine levels compared to non-diabetic controls, suggesting potential kidney dysfunction or impairment. Similarly, serum urea levels were also significantly elevated in diabetic cases compared to non-diabetic controls, supporting the possibility of impaired renal clearance. The substantial statistical significance of both serum creatinine and urea between the groups further substantiates this observation, with the median and IQR providing additional supporting evidence (Table 1).

An elevation in urea levels is indicative of kidney impairment or damage, while creatinine is an established marker of GFR. The simultaneous elevation of urea and creatinine, often alongside persistent hyperglycemia, is a hallmark of kidney damage [24]. A comparative study conducted in India (Biri et al., 2022) reported consistent findings, showing significantly higher blood urea (65.81 ± 12.12 mg/dL vs. 27.63 ± 5.32 mg/dL) and serum creatinine levels (1.89 ± 0.81 mg/dL vs. 0.84 ± 0.12 mg/dL) in diabetic patients compared to non-diabetics (*p* < 0.001) [12]. Our results align precisely with these findings, demonstrating higher levels of both creatinine and serum urea in our diabetic cohort compared to the controls (Table 1). The positive correlation observed between creatinine and serum urea was also more scattered in diabetic patients compared to the non-diabetic controls, where the association was less dispersed (Figure 1).

Diabetic nephropathy is a progressive, chronic condition that develops over many years, characterized by a gradual increase in urinary albumin excretion, hypertension, and elevated cardiovascular risk. It is marked by a declining GFR and can ultimately lead to end-stage kidney disease (ESKD) [23,24,25,26]. The duration of diabetes significantly influences the risk of diabetic nephropathy, particularly in type 1 diabetes [23]. Genetic factors also play a substantial role in susceptibility; for instance, the risk of nephropathy increases 4- to 8-fold in a sibling of a patient with type 1 diabetes who develops nephropathy, compared to siblings without the condition [27,28].

Our study ultimately confirms that elevated blood urea and serum creatinine levels indicate prolonged hyperglycemia, which causes irreversible damage to the kidney’s essential filtering units, the nephrons. Furthermore, high blood glucose levels impair the kidney’s ability to maintain fluid and electrolyte balance. The corresponding rise in serum creatinine and blood urea levels correlates directly with a decrease in GFR, unequivocally signaling compromised kidney function due to the diminished capacity to filter waste products from the blood [12]. Regular monitoring of these biomarkers is thus critical for the early detection and timely intervention necessary to effectively manage diabetic nephropathy and preserve long-term kidney health in diabetic patients.

In the future, further longitudinal research is required to track the changes in biomarkers over time, critically investigate the progression of diabetic nephropathy, and explore the impact of various interventions, such as improved glycemic control or blood pressure management, on renal function among diabetic patients. Furthermore, further investigation is essential to observe the association between renal biomarkers and microvascular dysfunction in diabetes. Recent evidence using laser speckle contrast analysis has demonstrated impaired skin microvascular function in patients with type 2 diabetes and its association with subclinical atherosclerosis [29]. Future studies could explore whether impaired renal function (e.g., creatinine, urea, and GFR alterations) parallels or predicts microvascular dysfunction, thereby providing a more integrated assessment of diabetic microvascular disease.

This retrospective study has certain limitations. A larger sample size could not be secured from the hospital register. Furthermore, we could not definitively confirm the complete health status of all non-diabetic participants. Serum urea level may also be elevated in a variety of clinical conditions other than kidney complications, which we could not confirm through the thorough check-up of each patient. Since the participants included in this study were included consecutively for a fixed period, variations in the number of populations, age, and gender between the case and control groups were present, which is one of the limitations of this study. Again, the study was limited by the inability to include a confirmed diabetic nephropathy group for a direct comparison of their biomarkers against our diabetic and non-diabetic cohorts. Additionally, the results may not be generalizable, as the data collection took place at a single center.

## 5. Conclusions

Serum creatinine, urea, and GFR are crucial prognostic markers and predictors for assessing renal damage in diabetic patients. The findings of this study confirm that elevated levels of serum creatinine and urea, coupled with lower levels of GFR, are strong indicators of impaired kidney function in individuals with diabetes. These analyses are highly valuable for enabling the monitoring and assessment necessary to manage diabetic nephropathy effectively, thereby contributing to improved long-term health outcomes for diabetic individuals. Further longitudinal research is warranted to comprehensively identify the association of these markers with other relevant indicators to better assess and prevent the initiation of nephropathy or other renal problems in both diabetic and non-diabetic populations.

## Figures and Tables

**Table 1:** Demographics of study participants.

General Characteristics	Total Participants		
Number	237		
Age (in years)	52.09 ± 15.72		
Female (*n*, %)	108, 45.57		
Male (*n*, %)	129, 54.43		
Diagnosis of chronic disease except diabetes	None		
Ethnicity	Asian		
Participant description	Diabetic patients (case)	Non-diabetic participants (control)	*p* value
Participants (*n*)	81	156	
Female (*n*, %)	43, 53.09	65, 41.67	
Male (*n*, %)	38, 46.91	91, 58.33	
Age (in years)	58.04 ± 15.69	48.86 ± 14.71	
OGTT (fasting) (mg/dL)	111.05 ± 13.73	87.81 ± 12.96	
OGTT (after 2 h) (mg/dL)	185.79 ± 44.76	102.13 ± 60.74	
HbA1c (%)	9.29 ± 7.45	5.27 ± 0.49	
Biomarkers (unit)			
Serum creatinine (mg/dL)	2.08 ± 2.27	0.95 ± 0.69	<0.001
Serum creatinine (median (IQR))	1.80 (1.15–2.20)	0.95 (0.67–1.19)	
Male (mg/dL)	2.16 ± 2.26	1.13 ± 0.69	
Female (mg/dL)	2.00 ± 2.26	0.68 ± 0.69	
Serum urea (mg/dL)	57.71 ± 38.75	31.79 ± 20.56	<0.001
Serum urea (median (IQR))	56.0 (41.0–75.0)	32.0 (23.0–43.0)	
Male (mg/dL)	53.34 ± 38.51	34.77 ± 20.49	
Female (mg/dL)	61.58 ± 38.51	27.66 ± 20.49	
GFR (ml/min/1.72 m^2^)	59.03 ± 34.20	96.72 ± 23.85	<0.001
GFR (median (IQR))	59.0 (35.0–78.0)	97.0 (78.0–116.0)	
Male (ml/min/1.72 m^2^)	58.55 ± 33.99	92.89 ± 23.77	
Female (ml/min/1.72 m^2^)	59.46 ± 33.99	103.07 ± 23.77	

## Data Availability

The original contributions presented in this study are included in the article/supplementary material. Further inquiries can be directed to the corresponding authors.

## Supplementary Materials

The following supporting information can be downloaded at: https://www.mdpi.com/article/10.3390/cimb48020167/s1.
