# Supplementary material for: Association of Serum Creatinine, Urea, and Glomerular Filtration Rate with the Progression of Diabetic Associated Kidney Complications: A Retrospective Case-Control Study
Source: Curr Issues Mol Biol. 2026 Feb 2;48(2):167. doi: 10.3390/cimb48020167 (PMC12939092; doi:10.3390/cimb48020167)
Supplement: Supplementary file 1 [file cimb-48-00167-s001.zip › cimb-4062337-supplementary.pdf]

Supplementary Files

Diabetic (case) group | **Non-diabetic (control) group**

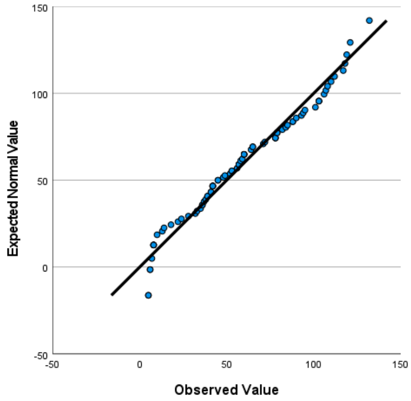

(a)

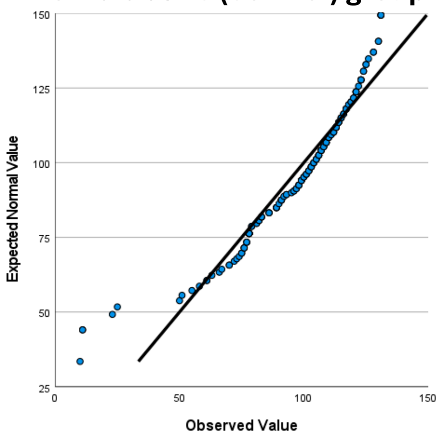

(b)

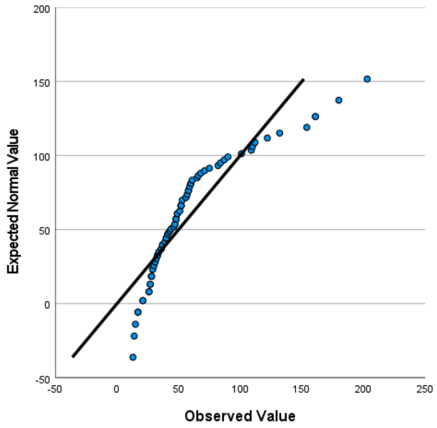

(c)

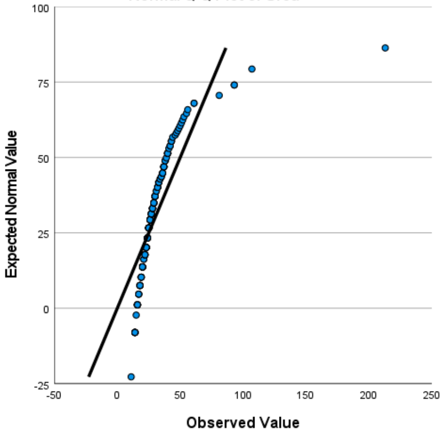

(d)

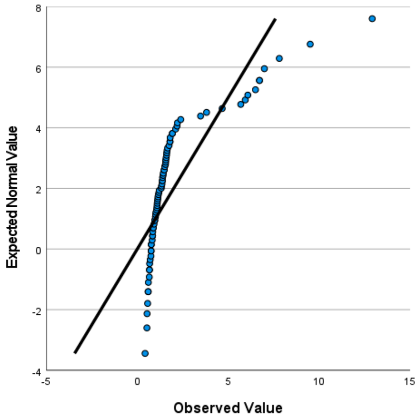

(e)

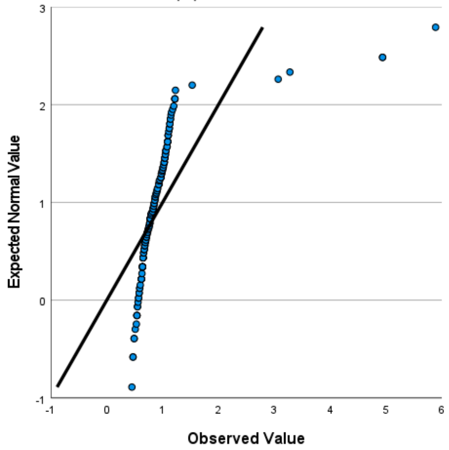

(f)

Supplementary Figure S1: Q-Q plot of (a) GFR, (c) urea, and (e) creatinine for diabetic patient (case) group and (a) GFR, (c) urea, and (e) creatinine for non-diabetic (control) group.

Diabetic (case) group | **Non-diabetic (control) group**

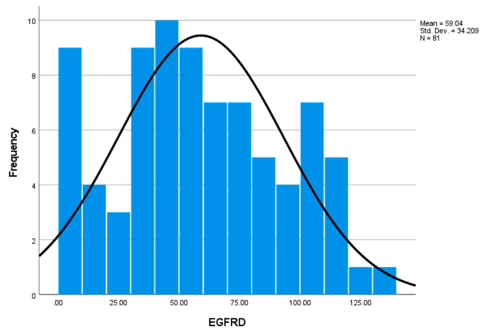

(a)

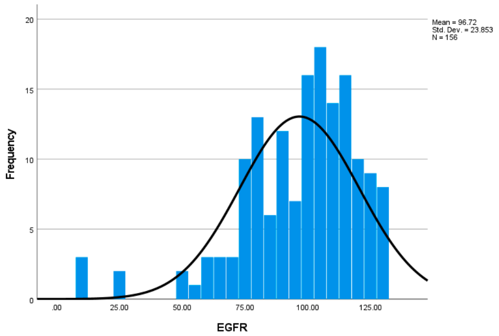

(b)

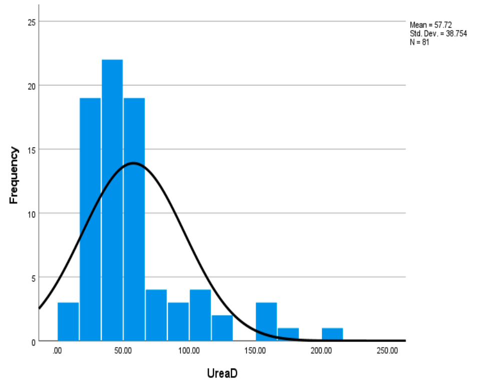

(c)

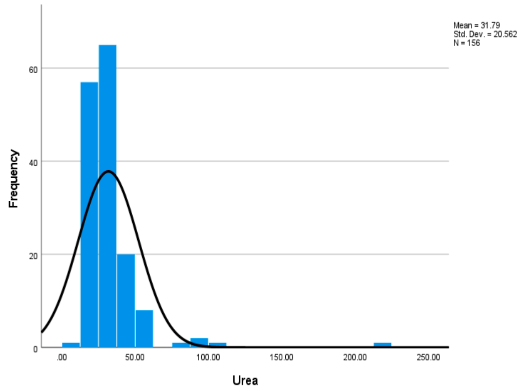

(d)

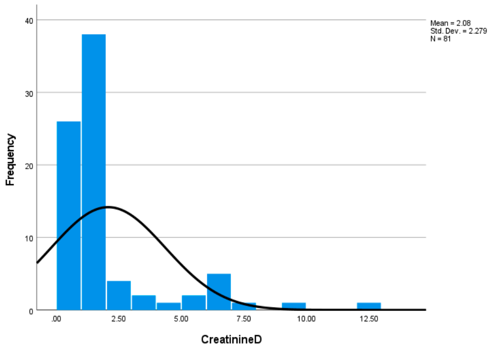

(e)

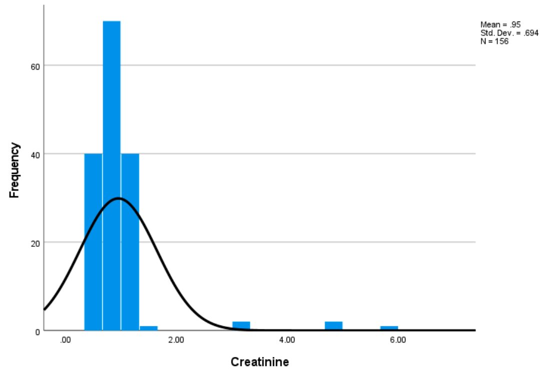

(f)

Supplementary Figure S2: Histogram of (a) GFR, (c) urea, and (e) creatinine for diabetic patient (case) group and (a) GFR, (c) urea, and (e) creatinine for non-diabetic (control) group.

ANOVA[a]

Model		Sum of Squares	df	Mean Square	F	Sig.
1	Regression	6.346	3	2.115	.747	.527[b]
	Residual	217.978	77	2.831		
	Total	224.324	80			

a. Dependent Variable: HBA1cD

b. Predictors: (Constant), CreatinineD, EGFRD, UreaD

Coefficients[a]

Model		Unstandardized Coefficients		Standardized Coefficients	t	Sig.
		B	Std. Error	Beta		
1	(Constant)	8.292	.900		9.208	.000
	EGFRD	.002	.009	.035	.197	.845
	UreaD	.004	.009	.092	.418	.677
	CreatinineD	.082	.148	.111	.551	.583

a. Dependent Variable: HBA1cD

(a)

ANOVA[a]

Model		Sum of Squares	df	Mean Square	F	Sig.
1	Regression	.727	3	.242	.996	.396[b]
	Residual	36.979	152	.243		
	Total	37.707	155			

a. Dependent Variable: HBA1c

b. Predictors: (Constant), EGFR, Urea, Creatinine

Coefficients[a]

Model		Unstandardized Coefficients		Standardized Coefficients	t	Sig.
		B	Std. Error	Beta		
1	(Constant)	5.712	.341		16.765	.000
	Urea	-.004	.003	-.156	-1.301	.195
	Creatinine	.005	.094	.007	.051	.959
	EGFR	-.004	.003	-.183	-1.424	.157

a. Dependent Variable: HBA1c

(b)

Supplementary Figure S3: Reports of multivariate analyses of the data of three biomarkers GFR, urea, and creatinine with corresponding HbA1c value of (a) diabetic (case) and (b) non-diabetic (control) group.
